# Tools Allowing Independent Visualization and Genetic Manipulation of *Drosophila melanogaster* Macrophages and Surrounding Tissues

**DOI:** 10.1534/g3.117.300452

**Published:** 2018-01-10

**Authors:** Attila Gyoergy, Marko Roblek, Aparna Ratheesh, Katarina Valoskova, Vera Belyaeva, Stephanie Wachner, Yutaka Matsubayashi, Besaiz J. Sánchez-Sánchez, Brian Stramer, Daria E. Siekhaus

**Affiliations:** * The Institute of Science and Technology Austria, 3400 Klosterneuburg, Austria; † Randall Division of Cell and Molecular Biophysics, King’s College London, SE1 1UL, United Kingdom

**Keywords:** macrophage, plasmatocyte, hemocyte, FACS, imaging, Genetics of Immunity

## Abstract

*Drosophila melanogaster* plasmatocytes, the phagocytic cells among hemocytes, are essential for immune responses, but also play key roles from early development to death through their interactions with other cell types. They regulate homeostasis and signaling during development, stem cell proliferation, metabolism, cancer, wound responses, and aging, displaying intriguing molecular and functional conservation with vertebrate macrophages. Given the relative ease of genetics in *Drosophila* compared to vertebrates, tools permitting visualization and genetic manipulation of plasmatocytes and surrounding tissues independently at all stages would greatly aid a fuller understanding of these processes, but are lacking. Here, we describe a comprehensive set of transgenic lines that allow this. These include extremely brightly fluorescing mCherry-based lines that allow GAL4-independent visualization of plasmatocyte nuclei, the cytoplasm, or the actin cytoskeleton from embryonic stage 8 through adulthood in both live and fixed samples even as heterozygotes, greatly facilitating screening. These lines allow live visualization and tracking of embryonic plasmatocytes, as well as larval plasmatocytes residing at the body wall or flowing with the surrounding hemolymph. With confocal imaging, interactions of plasmatocytes and inner tissues can be seen in live or fixed embryos, larvae, and adults. They permit efficient GAL4-independent Fluorescence-Activated Cell Sorting (FACS) analysis/sorting of plasmatocytes throughout life. To facilitate genetic studies of reciprocal signaling, we have also made a plasmatocyte-expressing QF2 line that, in combination with extant GAL4 drivers, allows independent genetic manipulation of both plasmatocytes and surrounding tissues, and GAL80 lines that block GAL4 drivers from affecting plasmatocytes, all of which function from the early embryo to the adult.

*Drosophila* plasmatocytes are well known for their immune functions in combatting bacteria, fungi, and viruses through phagocytosis and siRNA production (Braun *et al.* 1998; Elrod-Erickson *et al.* 2000; Lemaitre and Hoffmann 2007; Tassetto *et al.* 2017). Yet recent years have revealed the many ways in which they also play crucial roles in development and homeostasis, contacting and exchanging signals with surrounding cells. This has expanded the repertoire of functions that plasmatocytes are known to carry out to protect the organism; their patrolling serves not only to detect and destroy foreign invaders, but also to assess defects in endogenous cell states and stimulate corrective cellular responses. Many of the processes they affect and the molecular pathways they use to do so are conserved with vertebrate macrophages, making *Drosophila* plasmatocytes an excellent model system (Wynn *et al.* 2013; Ratheesh *et al.* 2015).

Plasmatocytes influence development in several different ways. They migrate widely in the embryo to phagocytose and thus clear cells that have undergone programmed cell death (Tepass *et al.* 1994; Zhou *et al.* 1995; Franc *et al.* 1996). As they move, plasmatocytes secrete extracellular matrix (ECM) components, which assemble into a stable basal lamina whose presence affects later steps in development (Fessler and Fessler 1989; Olofsson and Page 2005; Martinek *et al.* 2008; Matsubayashi *et al.* 2017). This effect can occur by the ECM providing a substrate for cell movement or by binding Dpp, a BMP family member, and influencing its signaling (Olofsson and Page 2005; Bunt *et al.* 2010; Van De Bor *et al.* 2015). These developmental functions are conserved in vertebrates. Vertebrate macrophages also engulf apoptotic cells during development (Gouon-Evans *et al.* 2000; Leers *et al.* 2002), and show molecular conservation with *Drosophila* in some of the receptors they use to recognize dying cells (Franc *et al.* 1996; Fadok *et al.* 1998; Manaka *et al.* 2004; Greenberg *et al.* 2006; Kurucz *et al.* 2007; Wu *et al.* 2009). Vertebrate macrophages secrete the ECM component collagen (Schnoor *et al.* 2008), which can bind BMP family members (Vukicevic *et al.* 1994; Sieron *et al.* 2002).

Plasmatocytes are also crucial for maintaining the organism after it has formed. They alter responses to damage in the gut, regulating stem cell proliferation by secreting stimulatory factors (Ayyaz *et al.* 2015; Chakrabarti *et al.* 2016). Plasmatocytes kill tumor cells by expressing TNFα (Parisi *et al.* 2014), or stimulate their invasion if tumors also express activated Ras, through MMP1 induction by TNFα-induced JNK signaling (Cordero *et al.* 2010; Pérez *et al.* 2017). Plasmatocytes can even alter metabolism and aging; upon engulfing lipids, they induce JAK-STAT signaling in surrounding tissues, which modulates insulin sensitivity, hyperglycemia, fat storage, and lifespan (Woodcock *et al.* 2015). Conservation with vertebrates is seen for these processes as well. Vertebrate macrophages alter gut stem cell proliferation to promote regeneration; they may also use BMP to do so as BMP2 inducible kinase is upregulated in responding gut tissues (Pull *et al.* 2005). Vertebrate macrophages can promote tumor induction of MMPs and invasion by secreting TNFα (Hagemann *et al.* 2005). Finally, vertebrate macrophages also participate in an inflammatory response to obesity that leads to insulin insensitivity (Weisberg *et al.* 2003; Xu *et al.* 2003; Patsouris *et al.* 2008; Pollard 2009), as is seen in the *Drosophila* response to lipid ingestion (Woodcock *et al.* 2015). Thus, conservation is seen between vertebrates and *Drosophila* in the ways in which immune cells and surrounding tissues affect one another and the molecular pathways they use to do so.

The genetic power of *Drosophila melanogaster* can help elucidate how plasmatocytes regulate organismal development and homeostasis, and how tissues signal their state to plasmatocytes to induce responses. Yet such studies require tools that are lacking, ones that permit the live imaging or manipulation of plasmatocyte behavior along with the modulation and visualization of other cells. Here, we describe a set of tools designed to facilitate these approaches and demonstrate that they function at all stages of the *Drosophila* life cycle. These lines will thus greatly aid investigations of the manifold interactions of *Drosophila* plasmatocytes with other tissues from birth to death, enabling insights that can be relevant for vertebrate systems.

## Materials and Methods

### Cloning

Standard molecular biology methods were used, and all constructs were first tested for functionality by transfection into the plasmatocyte-like S2 cell line (Schneider 1972; Woodcock *et al.* 2015) (a gift from Frederico Mauri of the Knoblich laboratory at IMBA, Vienna) before injection into flies. Restriction enzymes *BSi*WI, *Pst*I, and *Asc*I were obtained from New England Biolabs (Frankfurt, Germany); *Xba*I and *Eco*RI were from Promega (Mannheim, Germany). PCR amplifications were performed with CloneAmp HiFi PCR Premix from Clontech’s European distributor Takara Bio Europe (Saint-Germaine en Laye, France) using a peqSTAR 2× PCR machine from PEQLAB (Erlangen, Germany). All Infusion cloning was conducted using an Infusion HD Cloning kit obtained from Clontech’s European distributor (see above); relevant oligos were chosen using the Infusion primer tool at the Clontech website http://bioinfo.clontech.com/infusion/convertPcrPrimersInit.do.

### Construction of srpHemo-3XmCherry

A 2.5 kb *Xba*I-*Eco*RI fragment, which contains three repeats of mCherry, was cloned from pJJH1295 (Bakota *et al.* 2012; Evans *et al.* 2014) (a gift from Jürgen Heinisch, Addgene plasmid #36914), into the multiple cloning site of pCaSpeR4 (a gift from Leonie Ringrose, IMBA, Vienna). Subsequently, a 4.3 kb fragment of the *srp* promoter was amplified from plasmid srpHemoA (Brückner *et al.* 2004) (a gift from K. Brückner) by PCR with the following primers:

5′-CGAGGTCGACTCTAGAAAATTTTGATGTTTTTAAATAGTCTTATCAGCAATGGCAA-3′.
5′-ACGAAGCTTCTCTAGATATGGGATCCGTGCTGGGGTAGTGC-3′.

This fragment was cloned upstream of the 3xmCherry fragment at the *Xba*I site by Infusion cloning to create DSPL172.

### Construction of srpHemo-H2A::3XmCherry

A 458 bp fragment containing the first 124 amino acids from histone H2A was amplified from pKS23b, a gift from Kristen Senti and Julius Brennecke at IMBA, using the following primers:

5′-AGAGAAGCTTCGTACGCGTACGATGTCTGGACGTGGAAAAG-3′.
5′-CGACCTGCAGCGTACGCGTACGGCCGCCGCCTCTAGACACTT-3′.

This fragment was placed by Infusion cloning at the *BSi*WI site of DSPL172, downstream of the *srp* promoter and upstream of the 3XmCherry fragment, with the linker sequence SRGGGRTRTLQV to create DSPL216.

### Construction of srpHemo-moe::3XmCherry

An 869 bp fragment from the *Moesin* cDNA SD10366 (DGRC) (Rubin *et al.* 2000) containing amino acids 370–646, and thus the ERM domain of the protein, was amplified by PCR using the following primers:

5′-AGAGAAGCTTCGTACGATGGACACCATCGATGTGCA-3′.
5′-CGACCTGCAGCGTACGCATGTTCTCAAACTGATCG-3′.

This fragment was cloned as above at the *BSi*wI site in DSPL172, downstream of the *srp* promoter and upstream of the 3xmCherry fragment, with the linker MRTLQVD.

### Construction of srpHemo-QF2

A 4.3 kb fragment containing the srpHemo promoter was amplified from the srpHemoA plasmid (Brückner *et al.* 2004) (a gift from K. Brückner) using the following primers:

5′-TTATGCTAGCGGATCCAAATTTTGATGTTTTTAAATAGTCTTATCAGCAAT GGCAA-3′.
5′-TGGCATGTTGGAATTCTATGGGATCCGTGCTGGGGTAGTGC-3′.

This fragment was used to replace the synaptobrevin promoter in the *nsyb-QF2* plasmid (Riabinina *et al.* 2015) (a gift from C. Potter). The synaptobrevin promoter was released by a digest with *Bam*HI and *Eco*RI and replaced by srpHemo using Infusion cloning.

### Construction of srpHemo-GAL80

A 4.3 kb fragment of the *srp* promoter was amplified from plasmid srpHemoA (Brückner *et al.* 2004) (a gift from K. Brückner) by PCR with the following primers:

5′-GCATGTCGACCTCGAGAAATTTTGATGTTTTTAAATAGTCTTATCAGCAATGGCAA-3′.
5′-CTCCCGGGTACTCGAGTATGGGATCCGTGCTGGGGTAGTGC-3′.

This fragment was cloned by infusion into the (w+) attB plasmid (a gift from Jeff Sekelsky, Addgene plasmid #30326) at the *Xho*I site to create DSPL237.

A 1307 bp fragment containing GAL80 was amplified with the following primers from pAC-GAL80 (Potter *et al.* 2010; a gift from Liqun Luo, Addgene plasmid #24346):

5′-CTTCTGCAAGGCGCGCCCAATCAAAATGGATTACAACAAAAGGAG-3′.
5′-CGGTGCCTAGGCGCGCCTACCGGTAGACATGATAAGATACATTGATG-3′.

This fragment was introduced into DSPL237, downstream of the *srp* fragment at the *Asc*I site, using Infusion cloning to create DSPL322.

### Drosophila melanogaster stocks

Flies were raised on standard agar, cornmeal, molasses, and yeast food containing 1.5% Nipagin bought from IMBA (Vienna, Austria). Adults were placed in cages in a Percival DR 36VL incubator maintained at 29° and 65% humidity, and embryos were collected on standard plates prepared in house from apple juice, sugar, agar, and Nipagin, and treated with yeast from Lesaffre (Marcq, France). This applies to all experiments except the QF2 movie, whose fly husbandry conditions are described below. *repo-GAL4* and *QUAS-CD8*::*GFP* were obtained from the Bloomington *Drosophila* Stock Center, *UAS-moe*::*mCherry* from P. Martin (Millard and Martin 2008), *hml-dsRed* from K. Brückner, and *srp-GAL4 UAS-2xeGFP* from R. Reuter.

### Embryo immunohistochemistry

Embryos were fixed with a standard 18.5% formaldehyde/heptane fix for 20 min followed by methanol devitellinization. mCherry embryos were visualized directly after fixation, rehydration, and mounting. *srp-moe*::*GFP* embryos were rehydrated and underwent antibody staining, using standard protocols and overnight incubation with a 1:500 dilution of GFP antibody (Aves Labs, Tigard, OR), followed (after washing) by incubation for 2 hr with a 1:500 dilution of Goat anti-Chicken Alexa Fluor 488 secondary (Invitrogen, Carlsbad, CA). Embryos to be stained with Lz Ab (DSHB, Iowa City, IA) were heat-fixed using standard protocols and incubated overnight with a 1:20 dilution of the antibody, followed after washing by incubation for 2 hr with a 1:500 dilution of Goat anti-Mouse Alexa Fluor 488 secondary antibody (Invitrogen). After washing, they were mounted in Vectashield Mounting Medium (Vector Labs, Burlingame) on 76 × 26 mm slides from Glasfabrik Karl Hecht (Sondheim, Germany) with 22 × 40 mm coverslips, No. 1 thickness (VWR International, Radnor, PA).

### Microscopy

Embryo images were taken with an Inverted LSM700 Confocal Microscope from Zeiss (Jena, Germany), using a Plain-Apochromat 20×/NA 0.8 Air Objective. Larvae and adult flies were imaged with a Leica M205 FA Stereo Microscope, a Leica Planapo 2.0× objective, and a Leica DFC3000G camera (Wetzlar, Germany). Larvae were anesthetized for 10–15 sec with a FlyNap Anesthetic Kit (ArtNr 173010, Carolina Biological Supply Company, Burlington, NJ), rinsed 2× in water, then examined under the stereomicroscope. Adult flies were anesthetized for 3 min in FlyNap, and then immediately examined under the stereomicroscope. For imaging on the confocal, larvae and adults were prepared as described, and then mounted in Halocarbon 200 oil (CatNr: 25073-100, Polysciences Inc., Warrington, PA) in a sandwich of a plastic frame, a YSI 5685 Membrane Kit 002 (ArtNr: 1518-9862, Yellow Springs Instrument Co., Yellow Springs, OH) and a cover glass (CatNr: 631-014724X50 mm, thickness 1.5, VWR) immediately prior to visualization.

### Macrophage cell counts

Embryos were analyzed at stage 15–16 for total plasmatocyte number using Imaris (Bitplane) by detecting all the plasmatocyte nuclei as spots.

### Time-lapse imaging

For the *srpHemo-H2A*::*3xmCherry* time-lapse movies, embryos were dechorionated in 50% bleach for 4 min, washed with water, and mounted in halocarbon oil 27 (Sigma) between a coverslip and an oxygen-permeable membrane (YSI), as described above. The anterior dorsolateral region of the embryo was imaged on an inverted multiphoton microscope (TrimScope II, LaVision) equipped with a W Plan-Apochromat 40×/1.4 oil immersion objective (Olympus). mCherry was imaged at 1100 nm excitation wavelengths, using a Ti-Sapphire femtosecond laser system (Coherent Chameleon Ultra) combined with optical parametric oscillator technology (Coherent Chameleon Compact OPO). Excitation intensity profiles were adjusted to tissue penetration depth and Z-sectioning for imaging was set at 1 µm for tracking. For long-term imaging, movies were acquired for 180–200 min with a frame rate of 40 sec. All embryos were imaged with a temperature control unit set to 28.5°.

For the *srp-QF2 QUAS-mCD8*::*GFP repo-GAL4 UAS-moe*::*mCherry* time-lapse movies, flies were left to lay eggs on grape juice/agar plates overnight at 25°. Embryos were dechorionated in bleach. Stage 15 embryos of the appropriate genotype were identified based on the absence of balancer chromosomes expressing fluorescent markers, and mounted in 10S Voltatef oil (VWR) between a glass coverslip and a gas-permeable Lumox culture dish (Greiner), as described previously (Milchanowski *et al.* 2004; Evans *et al.* 2010). Movie images were taken at room temperature every 15 min on an Ultraview spinning disk microscope (PerkinElmer) equipped with a 20× NA 0.5 Plan-Neofluar air objective. Maximum projection images were made from ∼40 µm of Z stacks taken every 3 µm. Image processing was done by using ImageJ.

For the *srpHemo-moe*::*3xmCherry* time-lapse movies, embryos were dechlorinated in bleach for 1:15 min, and stage 15 embryos were identified and mounted in a slide covered with a double-sided sticky tape, oriented ventrally, and covered with 10S Voltatef oil (VWR) and a glass coverslip, as described in Stramer *et al.* (2010). Movie images were taken at room temperature every 5 sec on a Zeiss LSM 880 microscope, using Airyscan and a 63×/1.40 Oil DIC objective. Maximum projection images were made from ∼17 µm of Z stacks taken every 1 µm. Image processing was done by ImageJ.

### Transgenic line production

The *srpHemo*-*GAL80* construct was injected into lines y^1^ M{vas-int.Dm}ZH-2A w*; *M{3xP3-RFP.attP}ZH-51D* (BL 24483) and y^1^ M{vas-int.Dm}ZH-2A w*; *M{3xP3-RFP.attP}ZH-86Fb* (BL 24749), obtained from Peter Duchek of IMBA, to produce inserts on the second and third chromosomes through C31-mediated integration (Bischof *et al.* 2007). Our *srpHemo-QF2* driver was injected into *yw*; *p[w3′*, *y+*, *attP16a* (Okulski *et al.* 2011) to produce an insert on the second chromosome. After injection, all male survivors were crossed to *w*; *Sp/CyO*; *PrDr/TM3Ser* virgins. After hatching, we screened for transformants based on eye color and crossed them again to *w*; *Sp/CyO*; *PrDr/TM3Ser* virgins to get rid of the integrase inserted on the X chromosome. We kept three transformants/landing site.

All other vectors were co-injected into *w^1118^* (BL-3605) using standard injection methods, along with a helper plasmid Δ2-3 (Robertson *et al.* 1988) that permits *P* element transposase expression. w^+^ transformants were selected and double balanced.

### qPCR

RNA was isolated from ∼50,000 mCherry-positive or mCherry-negative cells using an RNeasy Plus Micro Kit (QIAGEN), following the manufacturer’s protocol. Of the resulting RNA, 50 ng was used for cDNA synthesis using the Sensiscript RT Kit (QIAGEN) and oligo dT primers. A Takyon qPCR Kit (Eurogentec) was used to mix qPCR reactions based on the provided protocol, using the following primers:

mCherry: 5′-ACATCCCCGACTACTTGAAGC-3′ and 5′ ACCTTGTAGATGAACTCGCCG-3′

which were designed using Primer-BLAST (https://www.ncbi.nlm.nih.gov/tools/primer-blast/index.cgi?LINK_LOC=BlastDescAd).

Pvr: 5′-GTGACTTTGGTCTGGCTCG-3′ and 5′-GATTCCAGCGCCAGC-3′.
RhoL: 5′-CCTGAGCTATCCCAGTACCAA-3′ and 5′-ACCACTTGCTTTTCACGTTTTC-3′.
Drpr: 5′-TCCACCTATCGCATTAAACACC-3′ and 5′-ACAGTCCCTCACAATACGGTT-3′.
RpL32: 5′-AGCATACAGGCCCAAGATCG-3′ and 5′-TGTTGTCGATACCCTTGGGC-3′.

These four sets of primers were obtained from FlyPrimerBank (http://www.flyrnai.org/FlyPrimerBank). qPCR was run on a LightCycler 480 (Roche) and data were analyzed using LightCycler 480 Software and Prism.

### FACS analysis

Embryos were collected for 1 hr from adult *w*^−^; *srpHemo-3xmCherry* flies and aged for an additional 4 hr, all at 29°. Embryos collected from *w*^−^ flies were processed in parallel and served as a negative control. Embryos were dissociated using a procedure adapted from Estrada and Michelson (2008). Embryos were dechorionated with fresh 50% bleach for 5 min, thoroughly rinsed with water, and blotted on a dry towel. Next, 30 mg of embryos were transferred with a paintbrush into a dounce homogenizer. Subsequent procedures were carried out at 4° or on ice, and all the solutions were cooled. Homogenizers were filled with 10 ml of Seecof saline (6 mM Na_2_HPO_4_, 3.67 mM KH_2_PO_4_, 106 mM NaCl, 26.8 mM KCl, 6.4 mM MgCl_2_, and 2.25 mM CaCl_2_ at a pH of 6.8) and embryos were homogenized with 10 vertical strokes. The resulting suspensions from three homogenizers were collected into a 50 ml Falcon tube (Corning, NY) and centrifuged at 500 rpm for 6 min 30 sec to pellet tissue debris. The supernatant was collected into a separate 50 ml Falcon tube and centrifuged at 1250 rpm for 10 min to precipitate cells. The supernatant was discarded, and the cell pellet was resuspended in 20 ml RPMI media with 10% FBS, which was then split into two 10 ml Falcon tubes. Next, 1 ml of heat-inactivated FBS was slowly pipetted down to the bottom of each of the tubes, which were subsequently centrifuged at 1250 rpm for 10 min to separate out the dead cells that remained in the upper phase after centrifugation. The resulting cell pellet was resuspended in 500 ml of Schneider’s media with 25% (0.2 μM filtered) heat-inactivated FBS and 2 mM EDTA (to reduce calcium dependent adhesion and thus the formation of clumps). The cell suspension was filtered to remove cell clumps using a Falcon 12 × 75 mm Polystyrene tube with a cell strainer cap containing a 35 μm nylon mesh. The cells were analyzed or sorted using a FACS Aria III (BD) flow cytometer. Emission signals for mCherry (600LP, 610/20), dsRed (583/15), and near infrared (755LP, 780/60) were detected. Data were analyzed with FloJo (Tree Star) software. The cells from the dissociated negative control *w*^−^ embryos were sorted to set a baseline plot. A sample of the cells from the dissociated *srpHemo-3xmCherry* embryos was stained with 2 μg/μl Propidium Iodide and almost no dead cells were detected upon sorting. Macrophages from these same *srpHemo-3xmCherry* embryos were sorted based on mCherry fluorescence into 2 ml Eppendorf tubes with 50 μl of Schneider’s *Drosophila* media.

For each genotype, 15 third-instar larvae were collected directly from bottles with a brush. Prior to homogenization, they were rinsed in water to dislodge any fly food residue and kept on ice to immobilize them. For each genotype, eight pairs of male and female adult flies were collected after CO_2_ anesthesia in an eppendorf tube and kept on ice till homogenization. Homogenization of larvae and adults, and FACS analysis, proceeded as for the embryos above, except that the LIVE/DEAD Fixable Near-IR Dead Cell Stain kit (Thermo Fisher Scientific) was utilized on a sample according to the manufacturers’ instructions. Data shown is representative of FACS from three independent experiments.

### Data availability

All plasmids and *Drosophila* strains created in this study are available from the authors upon request. All strains are also available through the Bloomington *Drosophila* Stock Center.

## Results

### Direct fusion lines visualize plasmatocyte nuclei, the cytoplasm, and the cytoskeleton in the embryo

**Figure 1:**
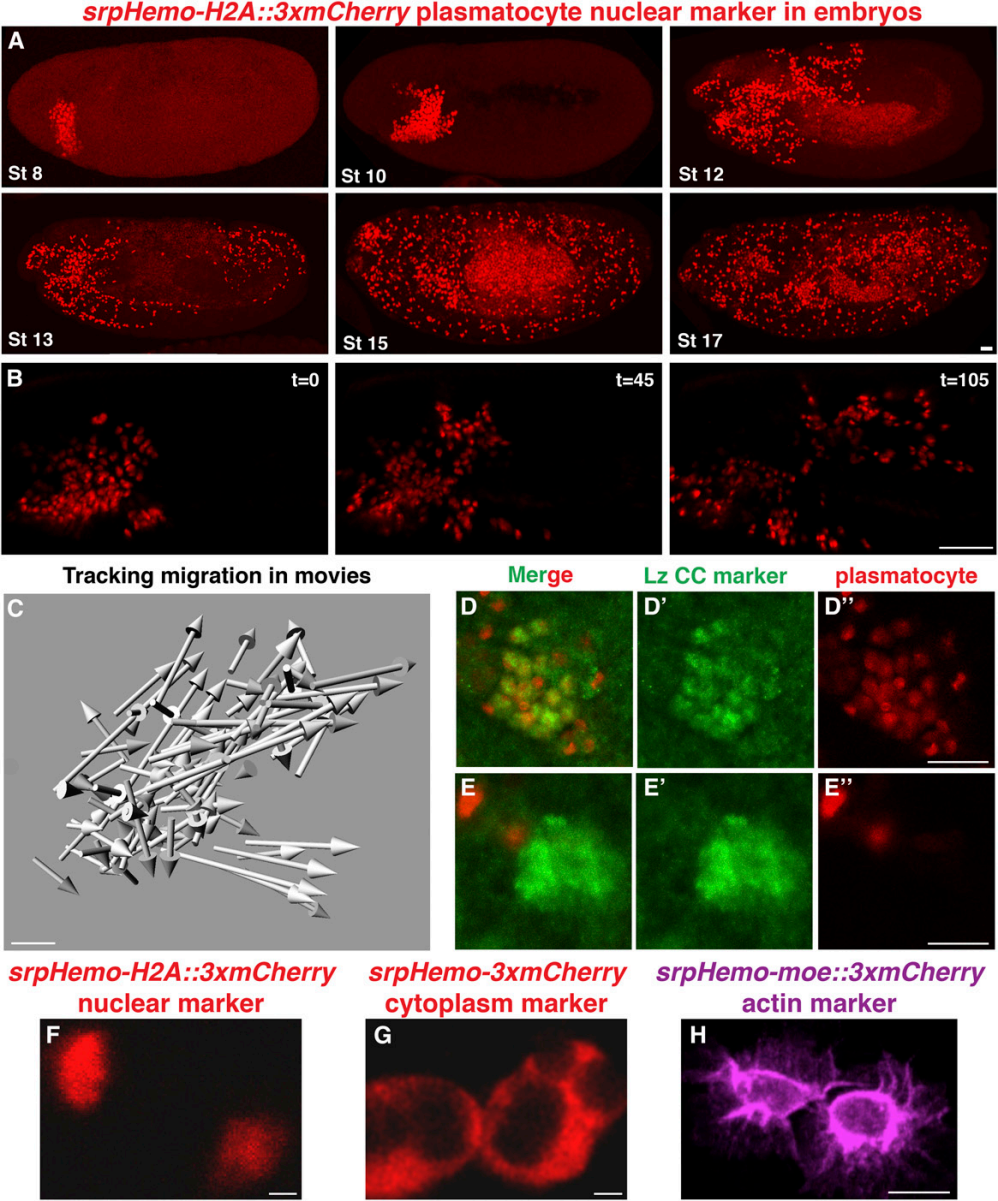
Direct fusion lines allow fluorescent visualization of plasmatocyte nuclei, the cytoplasm, or the cytoskeleton in embryos from stage (St) 8 onwards. (A) Fixed *srpHemo-H2A*::*3xmCherry* embryos display strong fluorescence in the nuclei of plasmatocytes starting from St 8 and continuing throughout embryogenesis. Embryonic St is indicated in the lower left of each panel. (B) Stills from a two-photon movie of a *srpHemo-H2A*::*3xmCherry* St 10–12 embryo illustrating the low level of endogenous autofluorescence in the yolk. Three successive time points are shown, with the intervening time in minutes indicated in the upper right of each panel. (C) Arrows indicating relative displacement of macrophage nuclei in 90 min of live imaging starting at St 10. (D and E) Close-ups of merged confocal images of *srpHemo-H2A*::*3xmCherry* embryos stained with Lz antibody (D’ and E’) as a marker of crystal cells (CC). We see mCherry expression in CC until St 14 (D”), but no longer at St 15 (E”). Live image of individual plasmatocytes visualized with a two-photon microscope with (F) *srpHemo-H2A*::*3xmCherry* or (G) *srpHemo-3xmCherry*, or (H) with a Zeiss confocal microscope from an *srpHemo-moe*::*3xmCherry* embryo demonstrating nuclear, cytoplasmic, or actin labeling, respectively. All embryos are positioned with anterior to the left and dorsal at the top. Scale bars correspond to 20 μM in (A), 40 μM in (B), 20 μM in (C and D), 2 μM in (F and G), and 10 μM in (H).

Visualizing plasmatocytes in fixed and live specimens is essential for understanding how these cells interact with surrounding tissues. Previous studies have labeled plasmatocytes by using various GAL4 drivers to activate UAS-reporters (Table 1; Evans *et al.* 2014). However, this approach prevents the simultaneous use of other GAL4 drivers to independently affect or image separate tissues. Direct fusions have been made of plasmatocyte-specific promoters to fluorescent proteins (Table 2; Evans *et al.* 2014), but none of these expressed at all stages of the life cycle. Additionally, the expression that many displayed was weak and, in some lines, was also present in large extraneous tissues, making live plasmatocyte detection and FACS analysis challenging. Therefore, we fused the *srpHemo* promoter that guides specific plasmatocyte expression in the embryo (Brückner *et al.* 2004) to three copies of mCherry (Shaner *et al.* 2004; Bakota *et al.* 2012), a red fluorescent monomer with a rapid maturation time, low photobleaching, and the ability to survive fixation with fluorescence intact. We also fused the first 124 amino acids of Histone H2A to mCherry, concentrating the signal in the nucleus to facilitate cell counting and tracking. There is little autofluorescence in the embryo in the red spectrum, and thus these *srpHemo-H2A*::*3xmCherry* lines displayed extremely brightly fluorescing plasmatocytes with little background starting at embryonic stage 8 and continuing through stage 17; the signal was still strongly visible after fixation with heat, formaldehyde, and paraformaldehyde—utilizing methanol, ethanol, or a hand-held needle to devitellinize—without any antibody staining required (formaldehyde/methanol is shown in Figure 1A). In contrast, the plasmatocyte fluorescence in the previously constructed *srp-moe*::*GFP* (Moreira *et al.* 2010) does not survive fixation (data not shown) and is weak when viewed live [Supplemental Material, Figure S1, A and B; asterisks in A show autofluorescent yolk granules as plasmatocytes only become evident live at stage 10 (data not shown)]. Upon staining with an antibody against GFP, plasmatocytes can be observed starting at stage 8 but are accompanied by strong extraneous expression in the amnioserosa (arrow in Figure S1, C and D), which is also seen live (arrow in Figure S1B) but not observed in live or fixed negative controls (data not shown). Thus, utilizing three copies of mCherry fused directly to the *srpHemo* promoter produces a plasmatocyte marker that is brightly visible in live or fixed embryos without antibody staining from early embryonic stages onwards.

**Table 1:** GAL4 Driver lines previously utilized for Plasmatocyte expression

Promoter Source	Tissue Expression of Reporter	Time of UAS-Reporter Expression in Plasmatocytes			References for Creation and Expression
		Embryo	Larva	Adult	
*serpent (srpHemo)*	P, LG in larva and adult PC	St 10–17	L1+, L2+/−, L3+/−	+	Brückner *et al.* (2004), Zaidman-Rémy *et al.* (2012)
*serpent (srp)*	P, LG, FB, embryonic midgut, amnioserosa, larval and adult PC, larval SG	St 10–17	L1+, L2+/−, L3+/−	+	Crozatier *et al.* (2004), Milchanowski *et al.* (2004), Avet-Rochex *et al.* (2010)
*croquemort*	P in adult, internal tissues	St 12–17	—	+	Olofsson and Page (2005) (embryo) Clark *et al.* (2011) (adult)
*peroxidasin*	P, LG from L2 on, in larva and adult PC, weak FB in L3	St 12–17	L1-L3	++	Stramer *et al.* (2005) (embryo) Stofanko *et al.* (2008) (larva) Ghosh *et al.* (2015) (adult)
*glial cells missing*	P, lateral glia	St 10–17	—	—	Bernardoni *et al.* (1997), Olofsson and Page (2005), Avet-Rochex *et al.* (2010)
*hemese*	80% of circulating P, sessile P, sections of midgut, SG	—	L3	—	Zettervall *et al.* (2004)
*hemolectin*	P, LG	—	L2, L3	+	Avet-Rochex *et al.* (2010), Sinenko and Mathey-Prevot (2004), Woodcock *et al.* (2015) (adult)
*collagen*	P, LG cortical zone, and FB at all stages	St 13–17	L1–L3	+	Asha *et al.* (2003), Avet-Rochex *et al.* (2010)
*singed*	P	St 11–17	—		Zanet *et al.* (2012)
*eater*	P, LG	—	L3	—	Tokusumi *et al.* (2009)

UAS, upstream activating sequence; P, Plasmatocytes; LG, lymph gland; PC, Pericardial cells; St, stage; FB, Fat Body; SG, salivary gland.

**Table 2:** Direct fusion lines for plasmatocyte visualization: previously published and described in this paper

Promoter Source-Reporter Utilized	Reporter Utilized: Tissue Expression	Time and Level of Reporter Expression in Plasmatocytes			References for Creation and Expression
		Embryo	Larva	Adult	
Previously published					
* hemolectin-DsRed*	P, CC, LG	—	+++L2–L3	+/−	Makhijani *et al.* (2011)
* hemolectin-DsRed*::*nls*	P, CC, LG	—	+++L2–L3	+/−	Clark *et al.* (2011), Makhijani *et al.* (2011)
* eater-DsRed*	P	—	+/−L3	—	Tokusumi *et al.* (2009), Ayyaz *et al.* (2015)
* eater-GFP*	P	—	+/−L3	—	Sorrentino *et al.* (2007)
* serpent-moe*::*GFP*	P, LG, in embryo amnioserosa	+	+/−L1–EL3	—	Moreira *et al.* (2010), Razzell *et al.* (2013)
This paper					
* srpHemo-3xmCherry and derivatives*	P, CC until embryonic stage 15, cortical zone of LG,	+++	+++	+++	This paper
	PC in second and third-instar larvae and adult, SGS from embryonic stage 16 till LL3	St 8–17	L1–L3		

P, Plasmatocytes; CC, Crystal Cells; LG, lymph gland; SGS, Stomatogastric nervous system; PC, Pericardial cells; St, stage.

We demonstrated the effectiveness of our *srpHemo-H2A*::*3xmCherry* nuclear line for tracking in live embryos by making two-photon movies of plasmatocyte migration from the head into the germband in stage 10–12 embryos (File S1) (Figure 1B). There was much less autofluorescence at the 1100 wavelength used for mCherry than at the 980 nm used for eGFP in the yolk and, particularly usefully, in the vitelline membrane, where absorption of laser energy through autofluorescence at 980 nm frequently leads to membrane rupture and death of the embryo during movie acquisition. The brightness of the mCherry signal also permitted the use of low laser power for effective imaging and thus less photobleaching. Analysis of plasmatocyte displacement based on tracking the nuclei with Imaris software revealed distinct paths of migration within the anterior, corresponding to the different directions ultimately chosen (Figure 1C). Efficient localization of *srpHemo-H2A*::*3xmCherry* to the nucleus (Figure 1F) also permitted easy determination of total plasmatocyte cell counts from confocal images using Imaris; we detected 592 ± 48 cells (*n* = 24) by analyzing wild-type stage 16 embryos, somewhat less than the 700 previously counted at stage 11 based on an antibody marker (Tepass *et al.* 1994). Thus, the nuclear-localized mCherry permits automated plasmatocyte tracking and counting, and eliminates many of the problems that occur with live two-photon imaging of GFP.

We assessed if our *srpHemo-H2A*::*3xmCherry* line also directs expression in crystal cells. These cells are born along with plasmatocytes from the mesoderm, migrate to a location around the proventriculus, and remain there during embryogenesis (Lebestky *et al.* 2000). In larvae and adults, they mobilize to enhance melanization in response to wounds or wasp egg infection (Galko and Krasnow 2004; Dudzic *et al.* 2015). We used an antibody recognizing Lozenge, a crystal cell marker, and observed colocalization with *srpHemo-H2A*::*3xmCherry* (Figure 1, D–D”) through much of embryogenesis, but by stage 15 (Figure 1, E–E”) no *mCherry* colocalization was detected. We also observed extraneous expression in the stomatogastric nervous system starting at stage 16/17 (data not shown). Given that there are 35 crystal cells (Milchanowski *et al.* 2004) and that we detect ∼600 total cells, we conclude that 94% of all embryonic cells labeled with *srpHemo-H2A*::*3xmCherry* before stage 15 are plasmatocytes.

We further created *srpHemo-3xmCherry* lines to produce plasmatocytes with a labeled cytoplasm (Figure 1G and Figure S1, E and E’), which are useful for studies examining direct contact of plasmatocytes with other tissues as well as their phagocytosis of pathogens and apoptotic cells. To visualize polymerized actin in plasmatocytes during studies of migration, we fused the mCherry with the C-terminal part of moesin that had been previously used to detect actin (Edwards *et al.* 1997). In these actin-binding *srpHemo-moe*::*3xmCherry* lines, we could detect filopodial and lamellipodial extensions within the plasmatocytes in live and fixed embryos (Figure 1H and Figure S1, F and F’), and could make time-lapse movies of plasmatocyte actin dynamics (File S2). Although the expression is weaker than in the cytoplasmic version, plasmatocytes can still be easily seen from all of these lines in fixed heterozygous embryos (Figure S1, E–G’), which allows analysis of the heterozygous progeny that arise, for example, during RNAi crosses. Heterozygotes can also be used in live imaging (nuclear line is shown in File S3, movie stills in Figure S1, H and H’). These lines were inserted at random positions on the second and third chromosomes, and are viable as homozygous embryos. Thus, our lines fusing the *srpHemo* promoter to 3xmCherry, either on its own or combined with other protein domains, permitted easy visualization of either the cytoplasm, nuclei, or actin cytoskeleton of plasmatocytes in the embryo in multiple contexts.

### Direct fusion lines visualize plasmatocyte nuclei, the cytoplasm, and the cytoskeleton in larvae and adults

**Figure 2:**
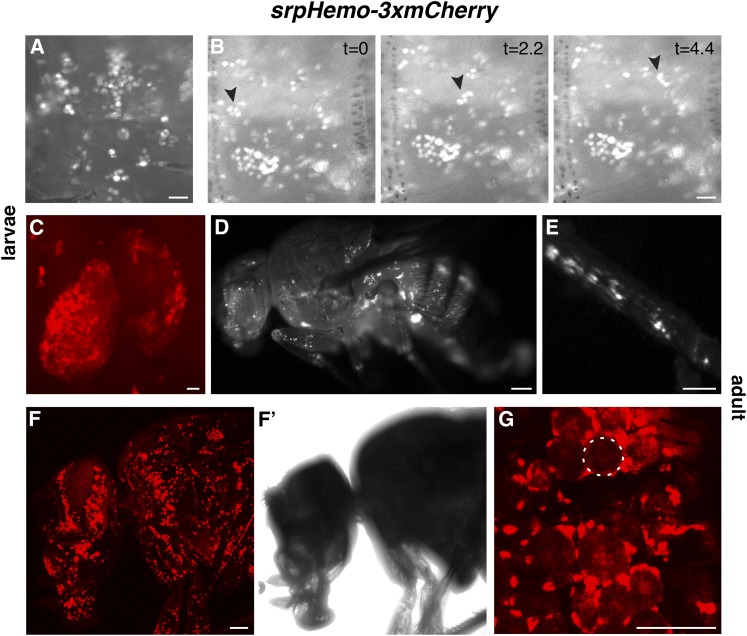
Direct fusion lines allow live imaging of plasmatocytes in larvae and adults. (A) Live image of plasmatocytes sitting on the body wall of a *srpHemo-3xmCherry* larva, viewed through the cuticle with a stereomicroscope. (B) Time-lapse imaging of plasmatocytes in a *srpHemo-3xmCherry* larva filmed through a stereomicroscope. Three successive time points separated by 2.2 sec each are shown. Arrowhead indicates a group of cells that float in the hemolymph while most other cells remain attached to the body wall. (C) Confocal image of labeling by *srpHemo-3xmCherry* third-instar larval lymph gland. (D) Live image of plasmatocytes in a *srpHemo-3xmCherry* adult and in a close-up of (E) the leg viewed through a stereomicroscope. (F–G) Live image of a *srpHemo-3xmCherry* adult viewed with a confocal microscope. (F) 3D projection of plasmatocytes in the head, proboscis, and thorax. (F’) transmitted light view of the adult fly imaged in (F). (G) Single confocal slice showing plasmatocytes encircling adult fat body cells (one indicated with white circle). Scale bars correspond to 500 μM in (A–C), 250 μM in (D and E), and 100 μm in (F–G).

These lines also permitted clear visualization of individual plasmatocytes in larvae and adults. In larvae, the characteristic pattern of resident plasmatocytes sitting in the body wall pockets (Makhijani *et al.* 2011) was most easily evident live through a stereomicroscope for the cytoplasmic 3xmCherry (Figure 2A), although it was also visible in the nuclear- and actin-localized forms (Figure S2, A and B). Individual plasmatocytes from the *srpHemo-3xmCherry* lines were also visible in these conditions floating in the hemolymph (Figure 2B and File S4), thereby allowing detection of the fluid flow. We frequently observed clusters of floating plasmatocytes adhering to a darker nonfluorescing droplet (most visible in File S4 when examining the cells indicated with an arrow in Figure 2B). The cortical zone of the third-instar larval lymph gland was labeled by mCherry (Figure 2C) along with 40 pericardial cells, pairs of large (50 μM) oval cells in a repeating pattern along the dorsal vessel that allow the heartbeat to be easily visualized (Figure S2C and File S5) (Das *et al.* 2007). We also observed this pericardial fluorescence in two other lines that visualize plasmatocytes, *srpHemo-GAL4 UAS-GFP* (Brückner *et al.* 2004) and *pxn-GAL4 UAS-GFP* (Stramer *et al.* 2005), but not in *hml-DsRed* (Makhijani *et al.* 2011) (data not shown). Expression was also seen in the stomatogastric nervous system during larval stages (Figure S2D). In fixed or live larvae, plasmatocytes were also visible deep within the body, at depths of ≤130 μm with confocal imaging (Figure S2, E and E’ shows a fixed first-instar *srpHemo-3xmCherry* with a 3D projection of plasmatocytes). At adult stages, cytoplasmic- (Figure 2, D and E), nuclear-, and actin-targeted mCherry-labeled plasmatocytes (Figure S2, G–I) were visible in the head, thorax, and legs using a stereomicroscope. Confocal images of live *srpHemo-3xmCherry* adults detected plasmatocytes within the body, at depths up to 94 μm (Figure 2, F and F’). The discovery of plasmatocytes encircling cells in the fat body (Figure 2G) is particularly interesting given recent results demonstrating their role in regulating metabolism (Woodcock *et al.* 2015). While in larvae the *srpHemo-3xmCherry* signal is similar to that seen in third-instar *hml-DsRed* larvae (data not shown), in the adult the *srpHemo-3xmCherry* signal is much brighter (compare Figure S2, F’ and G’) and can be easily detected in heterozygotes of all constructs (Figure S2, G’–I’). This allows direct detection of the presence of the chromosome in adults, greatly facilitating crosses. *srp-moe*::*GFP*, the other direct fusion line expressed beyond a single stage (Table 1), is much weaker than *srpHemo-3xmCherry* in second- and early third-instar larvae (Figure S3, A–C), and not detectible in late third-instar larvae and adults even with a confocal microscope (Figure S3, D–K). Thus, the *srpHemo-3xmCherry* lines permit visualization of plasmatocytes in live and fixed samples without antibody staining from the embryo to the adult.

### FACS sorting from the embryo to the adult using the direct fusion cytoplasmic line

**Figure 3:**
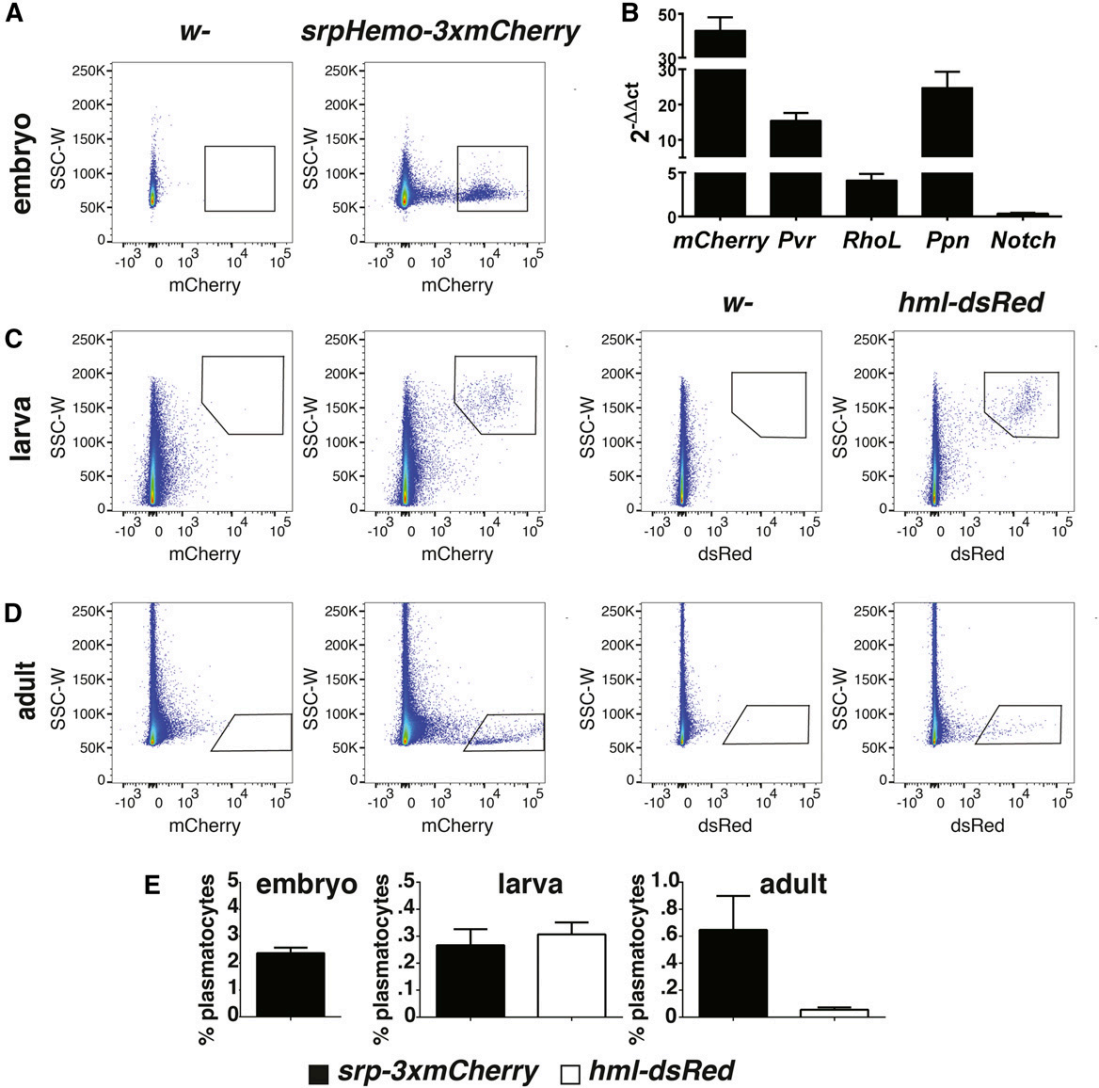
The direct fusion *srpHemo-3xmCherry* line allows Fluorescence-Activated Cell Sorting (FACS) of plasmatocytes from embryos, larvae, and adults. (A) FACS plot of Side Scatter (SSC) *vs.* mCherry fluorescence signal in cells obtained from control *w*- and *w*-; *srpHemo-3xmCherry* and embryos, showing strong separation of mCherry+ signal from the remaining cells. (B) quantitative PCR conducted on cDNAs prepared from RNA isolated from mCherry+- and mCherry−-sorted cells using primers recognizing the plasmatocyte markers *mCherry*, *Pvr*, *RhoL*, *Ppn*, and *Notch*. The data are normalized to results for the housekeeping gene *RpL32* and the graph shows the fold difference in signal observed between the mCherry+ (plasmatocytes) and mCherry− cells. (C and D) FACS plot of SSC *vs.* mCherry or DsRed fluorescence signal in cells obtained from *srpHemo-3xmCherry*, *hml-DsRed*, and control *w*-. In larvae (C), the two direct fusion lines show similar levels of fluorescent protein-positive plasmatocytes; however, in the adult (D), the number of DsRed+ plasmatocytes in *hml-DsRed* flies is strongly reduced when compared to the number of mCherry+ plasmatocytes in *srpHemo-3xmCherry*. (E) Quantification of plasmatocytes compared to total events detected during FACS analysis. Error bars in (B and E) represent SE of the mean. At least three independent experiments were conducted for each stage.

The *srpHemo-3xmCherry* line also facilitated purification of plasmatocytes by FACS. In stage 11 embryos, 2% of total cells from this line were mCherry-positive (Figure 3, A and E and Figure S4A). These cells were enriched for plasmatocyte markers such as *Pvr*, *Papilin*, and *RhoL* (Cho *et al.* 2002; Kramerova *et al.* 2003; Siekhaus *et al.* 2010), as assessed by qPCR (Figure 3B), but not for the broadly expressed gene *Notch* (Hartley *et al.* 1987), thus identifying the mCherry^+^ cells as plasmatocytes. We compared this line to the other extant direct fusion line in the red spectrum, *hml-dsRed*, which turns on in second-instar larvae. According to modENCODE data on FlyBase (http://flybase.org/reports/FBgn0029167.html), *hemolectin* is moderately expressed in LL3, almost absent in pupa, and shows low expression in adults, particularly females. We did not analyze *srp-moe*::*GFP* as it had strong extraneous expression in the embryo (Figure S1, B–D), was weak in second-early third-instar larvae, and showed no expression in late third-instar larvae and adults (Figure S3, A–K). The relative number of plasmatocytes was similar in *srpHemo-3xmCherry* and *hml-DsRed* in third-instar larvae (Figure 3, C and E), but we detected ≥10 times more fluorescent-positive plasmatocytes in *srpHemo-3xmCherry* than *hml-DsRed* adults (Figure 3, D and E), consistent with microscopic examination (Figure S2, F–G’), indicating very weak expression of *hml-DsRed* at this time. Using *srpHemo-3xmCherry*, we identified 0.25 and 0.6% of total cells as plasmatocytes in larvae and adults, respectively (Figure 3E and Figure S4, B and C). Thus, these *srpHemo*-derived constructs permit *in vivo* visualization and efficient FACS sorting, and analysis of plasmatocytes from the embryo to the adult independent of GAL4-based expression, unlike any other extant direct fusion line.

### QF2 lines allowing genetic manipulation of plasmatocytes from the embryo to the adult

**Figure 4:**
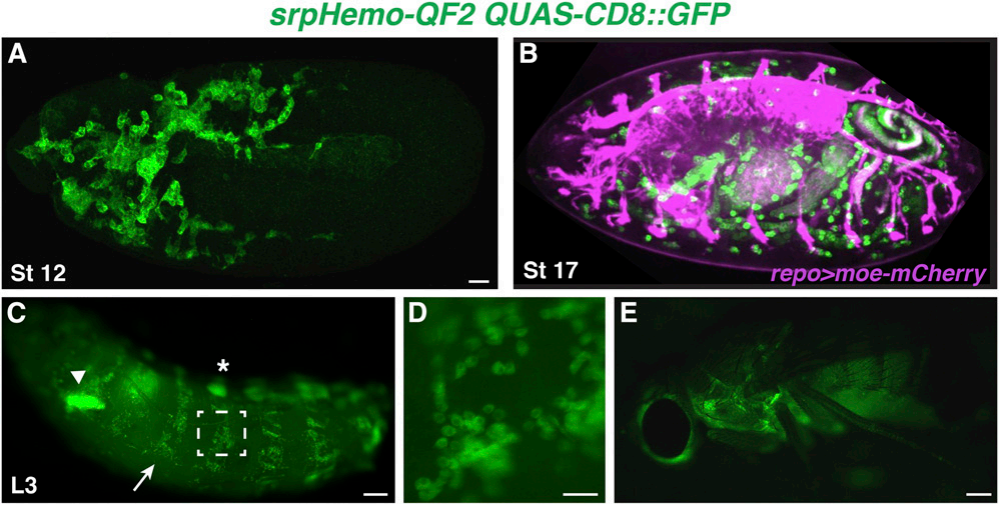
*srpHemo-QF2* enables independent genetic manipulation of plasmatocytes and surrounding tissues in the embryo to the adult. (A) Confocal image of fixed stage (St) 12 *srpHemo-QF2 QUAS-CD8*::*GFP* embryo showing QF2 dependent expression in plasmatocytes. (B) Still from live imaging with a spinning disc microscope of a *srpHemo-QF2 QUAS-mCD8-GFP/+*; *repo-GAL4 UAS-moe*::*mCherry/+* embryo demonstrating independent genetic control of plasmatocytes (in green) and the central nervous system (in purple). Anterior is to the left and the ventral side is up. (C–E) Stereomicroscope images of live samples. (C) *srpHemo-QF2 QUAS-CD8*::*GFP* third-instar larva showing expression in plasmatocytes (arrow), fat body (arrowhead), and cells along the dorsal vessel (asterisk). (D) Close up of plasmatocytes sitting on the body wall viewed through the cuticle from the region indicated in white box in (C). (E) *srpHemo- QF2 QUAS-CD8*::*GFP* adult. Anterior is to the left in all, dorsal is up in (A), (C–E), ventral is up in (B). Scale bars correspond to 20 μM in (A and B) and 500 μM in (C–E).

To permit genetic manipulation of plasmatocytes along with separate modulation of other tissues, we have taken advantage of the Q system (Potter *et al.* 2010; Potter and Luo 2011) and a nontoxic variant of the relevant transcription factor called QF2 (Riabinina *et al.* 2015). Our *srpHemo-QF2* driver integrated at the attP16a landing site on the second chromosome can control the expression of QUAS constructs such as *QUAS-CD8*::*GFP* in plasmatocytes (Figure 4A), starting at embryonic stage 10. We additionally observed lower-level expression from *srpHemo-QF2* either in the amnioserosa, mesoderm, and/or in punctate cells in the germband ectoderm in 11% of embryos (Figure S5, A and B). As QF2 does not bind to UAS sites, it can be combined with the known large repertoire of GAL4 drivers, which can then independently drive UAS constructs in other tissues. We illustrate this capability by combining *srpHemo-QF2 QUAS-CD8*::*GFP* with *repo-GAL4 UAS-moe*::*mCherry* to simultaneously label plasmatocytes and the embryonic nervous system (Figure 4B and File S6). In the larval stage, we see *srpHemo-QF2*-dependent expression detectable with a stereomicroscope again in the circulating and resident plasmatocyte population at the body wall during all larval stages (Figure 4, C and D), and in the third-instar larval lymph gland as well as pericardial cells (data not shown). Extraneous expression is seen in a subset of the fat body (arrowhead in Figure 4C). Plasmatocyte expression continues into the adult, which can be detected with a stereomicroscope (Figure 4E). Thus, the *srpHemo-QF2* line permits the independent visualization or genetic modification of plasmatocytes and surrounding tissues.

### GAL80 line blocking GAL4 action in plasmatocytes from the embryo to the adult

**Figure 5:**
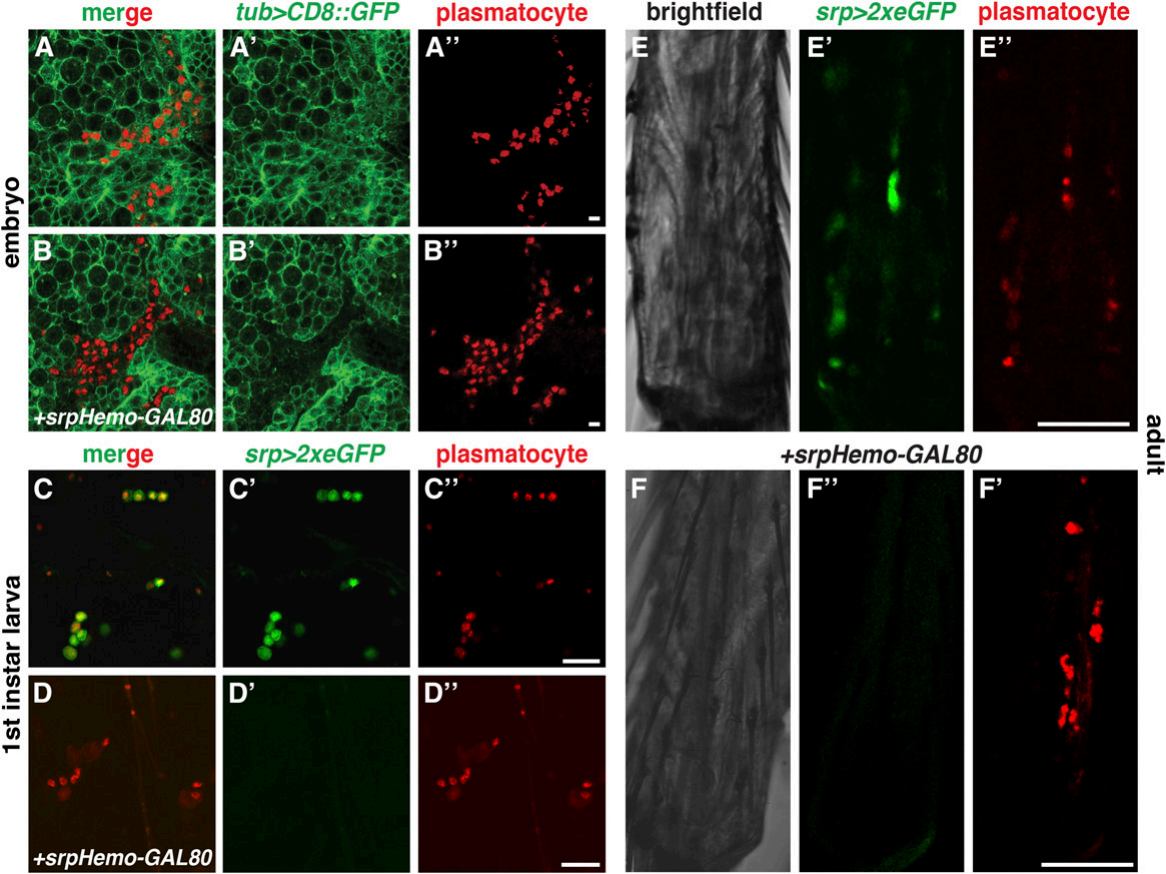
*srpHemo-GAL80* blocks the effect of GAL4 drivers on plasmatocytes. (A–F) Confocal images of fixed (A and B) and live (C–F) samples. (A–A”) Stage 11 *srpHemo-H2A*::*3xmCherry/+*, *tub-GAL4 UAS-CD8*::*GFP/+*, embryo showing the ubiquitous GAL4-dependent labeling of cell membranes by (A and A’) *UAS-CD8*::*GFP*, including in plasmatocytes labeled by the nuclear mCherry (A–A”). (B–B”) Stage 11 *srpHemo-H2A*::*3xmCherry srpHemo-GAL80/+*, *tub-GAL4 UAS-CD8*::*GFP/+*, embryo demonstrates that the expression of GAL80 in plasmatocytes labeled by nuclear mCherry (B”) leads to the suppression of CD8::GFP (B,B’). (C–C”) First-instar *srpHemo-H2A*::*3xmCherry/+*, *srp-GAL4 UAS-2xeGFP/+*, larva shows that plasmatocytes labeled by the nuclear mCherry also express cytoplasmic GFP. However, in a larva also carrying *srpHemo-GAL80*, the plasmatocytes (D) no longer express the GAL4-controlled GFP (D’ and D”). (E–E”) Legs of *srpHemo-H2A*::*3xmCherry/+*, *srp-GAL4 UAS-2xeGFP/+*, adults; GFP is expressed in plasmatocytes under GAL4 control (E’ and E”), but not in the presence of *srpHemo-GAL80* (F’ and F”). Scale bars correspond to 20 μm in (A and B), 20 μm in (C and D), and 50 μm in (E and F).

Finally, we wished to be able to genetically alter *Drosophila* using broadly expressed GAL4 drivers while not affecting plasmatocytes themselves. To this end, we utilized GAL80, which blocks the activity of GAL4 (Lee and Luo 1999), and created *srpHemo-GAL80* lines. This construct was integrated on the second and third chromosome at the split *white* attP landing sites at ZH-51D and ZH-86Fb, which contain 3xP3-RFP and can be recognized in larvae by the remaining landing site red fluorescence in the brain (Figure S6A), hindgut (Figure S6B), and intersegmental nerves (asterisk in Figure S6E”), and in the top of the head (Figure S6, C and C’) (Bischof *et al.* 2007) in the adult, aiding detection of the chromosome in crosses. Should this extraneous RFP be deleterious for planned experiments, it can be eliminated from the line by expressing cre recombinase. To demonstrate the use of this construct, we visualized plasmatocytes using the above-described *srpHemo-H2A*::*3xmCherry* and utilized the ubiquitous driver *tub-GAL4* to express *UAS-CD8*::*GFP* in the entire embryo (Figure 5, A–A”). The addition of *srpHemo-GAL80* was able to block *tub-GAL4*-based expression of *CD8*::*GFP* in plasmatocytes (Figure 5, B and B”), but did not affect any of the surrounding cells (Figure 5, B and B’). To visualize this effect in larvae and adults, we shifted to GAL4-based expression of GFP just in plasmatocytes using *srp-GAL4 UAS-2xeGFP*. We observed the same capacity of the *srpHemo-GAL80* to suppress the effect of GAL4 in plasmatocytes, resulting in no GFP expression in first- to third-instar larvae (compare Figure 5, C–C” to Figure 5, D–D” and Figure S6, D–D” to Figure S6, E–E”) and adults (compare Figure 5, E–E” to Figure 5, F–F”). We also noted that *srp-GAL4* at these stages labeled only a subset of the plasmatocytes visualized with *srpHemo-3xmCherry* (Figure S6, D and E). Thus, *srpHemo-GAL80* can insulate plasmatocytes from the effects of broadly expressed GAL4 drivers in the embryo, larva, and adult.

## Discussion

In recent years, plasmatocytes have been shown to be able to detect multiple physiological conditions, and produce adaptive and sometimes deleterious responses to them. Much of this work has focused on the signals sent from plasmatocytes to the surrounding tissues and the resulting effects (Ayyaz *et al.* 2015). To investigate the reverse aspect, namely how tissues signal to plasmatocytes and influence immune cell number or behavior, tools permitting the visualization or isolation of plasmatocytes in conditions where only surrounding tissues have been genetically altered are required. We have created three extremely bright lines that allow the easy detection of the plasmatocyte nucleus, cytoplasm, or actin cytoskeleton live or upon fixation from embryonic stage 8 until the adult in homozygotes and heterozygotes. The cytoplasmic line is particularly effective for FACS purification at all stages, facilitating quantitative assessment of the numbers of plasmatocytes and the levels of proteins expressed in them. This will also support next-generation sequencing analysis of the plasmatocyte transcriptome at many stages and eventually at the single-cell level. Our additional creation of *srpHemo-QF2* and *srpHemo-GAL80* facilitate targeted genetic manipulations in combination with other GAL4 drivers. Thus, we have produced a comprehensive set of tools permitting the analysis and genetic screening of plasmatocyte behaviors at all stages of the *Drosophila* life cycle.

Several of these new tools will permit experiments on plasmatocyte migration that were not feasible until now. Plasmatocytes are born from the mesoderm and start to migrate at embryonic stage 8, three stages and 3 hr before stage 11 when the previous visualization techniques using GAL4 and fluorescent reporters allowed their detection. Thus, the mechanisms that trigger the initiation of their movement, their coordination while they are in closer contact, or their choices to split into different paths (all of which occur prior to stage 10) have not been investigated. The extant direct fusion *srp-moe*::*GFP* line is weakly expressed in the embryo, absent in late larvae and adults, and utilizes a fluorophore whose activation and emission spectra is shared by many autofluorescent molecules in the fly. Thus, our *srpHemo* direct fusion lines that start expression at stage 8 will serve as the foundation for studies to address these migratory questions, with the nuclear line facilitating tracking and the actin labeling line aiding examination of the cytoskeletal underpinnings of this developmental movement. These lines will also aid investigations into the migration posited to underlie the final homing of plasmatocytes to their positions on the larval body wall, where they proliferate (Makhijani *et al.* 2011; Van De Bor *et al.* 2015), and to the dorsal clusters in the adult (Ghosh *et al.* 2015), which could shed light on resident macrophage homing in vertebrates.

The movement of plasmatocytes allows them to reach tissues where they are known to play important roles, responding to wounds (Stramer *et al.* 2005; Wood *et al.* 2006), engulfing dead cells (Tepass *et al.* 1994; Franc *et al.* 1996; Weavers *et al.* 2016a), promoting or killing tumors (Cordero *et al.* 2010; Parisi *et al.* 2014), regulating stem cell proliferation (Ayyaz *et al.* 2015; Van De Bor *et al.* 2015), and monitoring metabolism (Woodcock *et al.* 2015). The nature, though not the identity, of the cues that guide them to these tissues is somewhat understood for wounds (Razzell *et al.* 2013; Weavers *et al.* 2016b) and tumors (Pastor-Pareja *et al.* 2008), and remains completely unknown for the rest. Screens utilizing GAL4 expression of RNAi constructs in these tissues and monitoring plasmatocyte responses will be greatly aided by all three of our direct fusion lines, which are visible as heterozygotes. Such screens seeking to quantitatively examine effects on plasmatocyte proliferation throughout the organism should utilize FACS analysis and our *srpHemo-3xmCherry* line, which is effective from stage 8 to the adult. FACS analysis will detect changes in proliferation in both the lymph gland and the tissue-resident populations, as we see expression in plasmatocytes in both regions. If the chosen driver expresses broadly, our *srpHemo-GAL80* can be used to block the activity of GAL4 in plasmatocytes from stage 9 in the embryo to the adult and allow the RNAi screen to only affect surrounding tissues. How tissues and plasmatocytes signal back and forth to one another can be investigated using our *srpHemo-QF2* line in addition to extant GAL4 drivers to modulate the genetic behavior on both sides. If the process is only being investigated in L3 larvae and beyond, then the extant *hml-QF2* (Lin and Potter 2016) can be used (Table 3). Thus, these lines should allow the identification of new mechanisms underlying plasmatocyte migration, and regulatory interactions between plasmatocytes and surrounding tissues at all stages of the *Drosophila melanogaster* life cycle.

**Table 3:** QF2 and GAL80 lines: previously published and described in this paper

Promoter Source-Txion Factor	Tissue Expression	Time and Level of Effect in Plasmatocytes			Reference for Creation and Expression
		Embryo	Larva	Adult	
*hemolectin-QF2*	P, CC		+++L2–L3	+	Expression based on *hml-GAL4*, same promoter (see Table 1) Lin and Potter (2016)
*srpHemo-QF2*	P, CC until embryonic stage 15, LG, PC in third-instar larvae and adult, small patch in larval FB	+++	+++	+++	This paper
*srpHemo-GAL80*	P, CC until embryonic stage 15, LG, PC in larvae and adult, SGS from embryonic stage 16 till LL3	+++	+++	+++	This paper

P, Plasmatocytes; CC, Crystal Cells; LG, lymph gland; FB, Fat Body; SGS, Stomatogastric nervous system; PC, Pericardial Cells.

We hope that these reagents will also spur on new types of studies in the adult. The previously created *hml-DsRed* is visible in third-instar larvae, yet in adults *hml-dsRed* is hard to detect; our *srpHemo-3xmCherry* line (Figure 2, C–G and Figure 3D) thus enables experiments that were previously difficult. While plasmatocytes have been shown to regulate metabolism and affect aging (Woodcock *et al.* 2015), further investigations of how aging tissues signal to stimulate adaptive or deleterious plasmatocyte responses require direct visualization and FACS analysis of plasmatocytes in the adult. The role of other tissues in potentially influencing plasmatocyte responses to infection (Buchon *et al.* 2014) is another area that these lines could beneficially impact, by enabling screens as described above.

Given the wide range of processes *Drosophila* plasmatocytes have been shown to participate in, this set of tools will immediately prove useful to a broad number of scientists studying *Drosophila* development, aging, cancer, stem cells, wounds, immunity, and metabolism. Since plasmatocytes interact with tissues throughout the organism at all stages, these tools will also facilitate the discovery and investigation of many as yet unidentified regulatory processes. The genetic conservation observed between *Drosophila* and vertebrates strongly suggests that this future work will also prove beneficial for studies in higher organisms.

## Supplementary Material

Supplemental material is available online at www.g3journal.org/lookup/suppl/doi:10.1534/g3.117.300452/-/DC1.
